# Analysis of the association between high antioxidant diet and lifestyle habits and diabetic retinopathy based on NHANES cross-sectional study

**DOI:** 10.1038/s41598-024-62707-7

**Published:** 2024-05-24

**Authors:** Qincheng Qiao, Xingjian Liu, Wen Xue, Li Chen, Xinguo Hou

**Affiliations:** 1 Department of Endocrinology and Metabolism, Qilu Hospital of Shandong University, Jinan, 250012 Shandong People’s Republic of China; 2 Institute of Endocrine and Metabolic Diseases of Shandong University, Jinan, 250012 China; 3 Key Laboratory of Endocrine and Metabolic Diseases, Shandong Province Medicine and Health, Jinan, China; 4 Jinan Clinical Research Center for Endocrine and Metabolic Diseases, Jinan, China; 5 The First Clinical Medical College, Cheeloo College of Medicine, Shandong University, Jinan, China

**Keywords:** Endocrinology, Medical research

## Abstract

Oxidative stress plays a crucial role in increasing the risk of developing diabetic retinopathy (DR). The oxidative balance score (OBS) and the composite dietary antioxidant index (CDAI) are two tools for assessing the effects of diet and lifestyle on oxidative stress. The aim of this study was to investigate the association between OBS, CDAI and the occurrence of DR. After controlling for potential confounders, OBS was negatively associated with DR with an odds ratio (OR) of 0.976 and a 95% confidence interval (CI) of 0.956–0.996, suggesting that for every unit increase in OBS, the risk of DR was reduced by 2.4%. In contrast, the relationship between OBS and CDAI was not significant (P > 0.05), suggesting that it was OBS, not CDAI, that contributed to the reduced risk of diabetic retinopathy. After adjusting for potential confounders, OBS was negatively associated with DR (OR: 0.976; 95% CI 0.956–0.996), but this association was not found in CDAI (P > 0.05), suggesting that for every one-unit increase in OBS, there was a 2.4% reduction in the risk of developing DR. This study suggests that a diet and lifestyle high in OBS reduces the risk of developing DR, which provides a rationale for nutritional interventions to prevent DR.

## Introduction

Diabetic retinopathy (DR) is a microvascular complication of diabetes that severely affects the quality of life and socioeconomic status of patients and is the leading cause of vision loss among working-age adults in many countries^1,2^. It is estimated that 34.6% of people with diabetes worldwide have DR, and one in ten people with diabetes has vision-threatening DR^3^. Besides the impact on vision, the presence of DR also indicates an increased risk of systemic vascular complications^4^, which imposes a great burden on patients and society. Therefore, DR has become an urgent public health issue in modern society, and how to effectively prevent and treat DR remains a challenge. Several factors influence the onset and progression of DR, with oxidative stress identified as a significant risk element^5^.

The oxidative balance score (OBS) serves as a holistic indicator of a person’s level of oxidative stress, which assesses the oxidative balance of a subject’s lifestyle based on the intake of antioxidants and pro-oxidants^6,7^. OBS can reflect the diet and lifestyle that may lead to adverse health outcomes and is of great value in epidemiological studies, especially in chronic disease studies^8^. The combined dietary antioxidant index (CDAI) is a tool to assess an individual’s total dietary antioxidant intake (TAI), which includes six dietary antioxidants, namely vitamins A, C, and E, carotenoids, selenium, and zinc^9^. Previous studies have shown that CDAI is associated with specific inflammatory biomarkers^10^ and various health outcomes in the population^11^.

However, there are few studies on the relationship between OBS and CDAI and DR. Therefore, in this study, we used data from the National Health and Nutrition Examination Survey (NHANES) to explore the association between DR and OBS and CDAI, as well as the influence of different population characteristics. We aim to provide a nutritional and lifestyle basis for the prevention and intervention of DR.

## Method

### Survey description

The NHANES study protocols adhered to the ethical guidelines of the 1975 Declaration of Helsinki and received approval from the NCHS research ethics review board. Written informed consent was obtained from all participants. Detailed study designs and data from NHANES are publicly accessible at www.cdc.gov/nchs/nhanes/. This report complies with the strengthening the reporting of observational studies in epidemiology (STROBE) guidelines for cross-sectional studies.

### The study population

**Figure 1:**
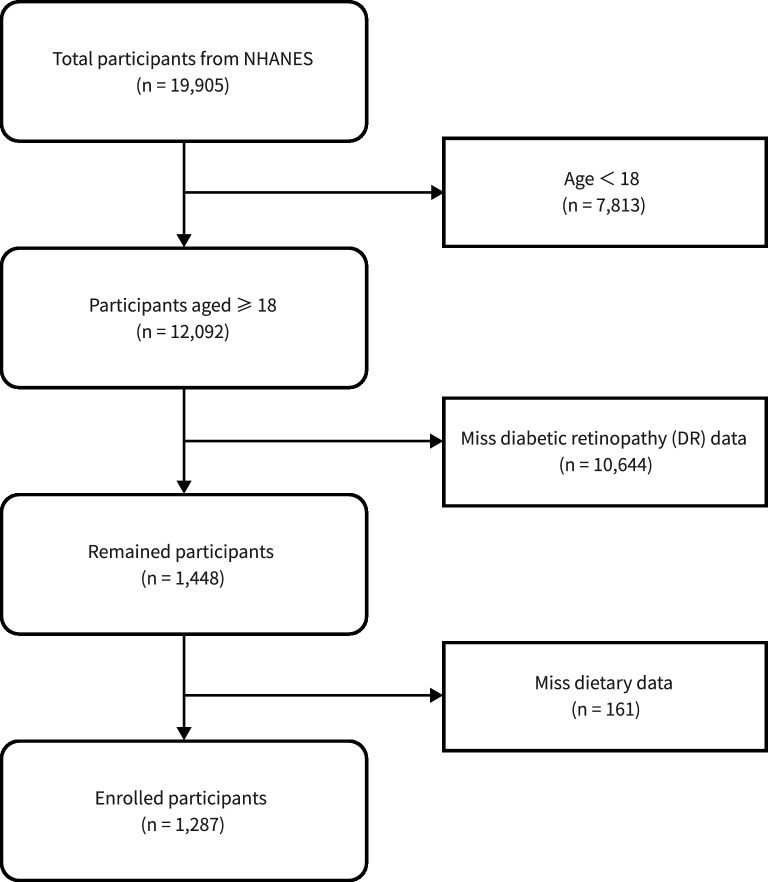
Flow diagram of the study population’s inclusion and exclusion criteria. 2007–2008, 2011–2012 National Health and Nutrition Examination Survey (NHANES).

The research incorporated subjects from NHANES, a cross-sectional study designed and executed by the National Center for Health Statistics (NCHS) aimed at evaluating the health and nutritional condition of both adults and children across the United States. The study used data from two 2 year NHANES study cycles in 2007–2008 and 2011–2012, which enrolled a total of 19,905 participants. Eligibility for the study was determined by participants being 18 years or older and having accessible data on diabetic retinopathy outcomes. The exclusion criteria were participants with fewer than 16 OBS component items and missing DR data^12^. In the end, the study encompassed 1287 qualified participants, as shown in Fig. 1. The NCHS ethics review committee granted approval for this research, and each participant gave their written consent, fully informed of the study’s scope.

### Exposure definitions

OBS stands for oxidative balance score, which is a measure of the antioxidant capacity of an individual or group. OBS consists of two components: dietary OBS and lifestyle OBS, which consist of 20 nutrients associated with oxidative stress, five of which are pro-oxidants and 15 of which are antioxidants. Dietary OBS includes 16 nutrients such as dietary fiber, carotene, riboflavin, niacin, vitamin B6, total folate, vitamin B12, vitamin C, vitamin E, calcium, magnesium, zinc, copper, selenium, total fat and iron. Lifestyle OBS includes four behavioral factors associated with oxidative stress, namely alcohol consumption, smoking, body mass index, and physical activity. OBS is calculated by adding scores for these factors, with higher scores indicating greater antioxidant capacity. The calculation of OBS is based on earlier studies^7^. CDAI stands for combined dietary antioxidant index, which is a measure of dietary antioxidant intake. CDAI includes six major dietary antioxidants, namely vitamins A, C, and E, carotenoids, selenium, and zinc. CDAI is calculated by subtracting the average of the intake of these antioxidants, then dividing by the standard deviation for standardization, and finally adding the standardized values to arrive at the final CDAI score^11^.

For a detailed understanding of the dietary habits of the U.S. population, NHANES operates as a collaborative nutrition evaluation effort between the National Center for Health Statistics (NCHS) and the USDA’s food survey research group (FSRG). NHANES’ nutritional analysis includes conducting 24 h dietary recall interviews in person with individuals across various age groups, carried out by skilled interviewers specializing in dietary assessments. In addition, NHANES collected lifestyle data on participants’ alcohol consumption, smoking, body mass index, and physical activity, which were also incorporated into the analysis of indicators related to OBS. Alcohol consumption is the average number of alcoholic beverages of any type consumed per day in the past year. The main metabolite of nicotine is cotinine^12^, which can be detected in serum, urine, or saliva. Cotinine serves as a biomarker for both active and passive smoking due to its longer blood half-life compared to nicotine. In this research, plasma cotinine was chosen to measure exposure levels quantitatively. The term “Activity Intensity (AP)” denotes the weekly total of physical activity, calculated by multiplying the metabolic equivalent (MET) score by both the frequency and duration of each physical activity^13^.

### Outcome definitions

For the definition of type 2 diabetes, we considered a combination of ADA criteria and previous NHANES studies: a fasting plasma glucose (FPG) concentration above 126 mg/dL (7.0 mmol/L), a 2 h plasma glucose concentration over 200 mg/dL (11.1 mmol/L), or a hemoglobin A1C (HbA1c) percentage of 6.5% or higher in an oral glucose tolerance test (OGTT) indicates the thresholds^14,15^. DR is reported as a binary outcome, confirmed by a physician informing the respondents that diabetes was affecting their eyes^16^.

### Covariate definitions

In our current study, in order to clarify the influence of potential confounders, we selected the following covariables when studying OBS: age, gender, race, household income, education level, low-density lipoprotein (LDL), high-density lipoprotein (HDL), triglycerides (TG), and HbA1c. When studying CDAI, we selected the following covariates: age, gender, race, household income, education level, alcohol, BMI, hypertension, smoking, blood fats (LDL, HDL, TG), and HbA1c. Information was collected from NHANES household interviews on a variety of demographic and health-related factors, including age, gender, race, education level, household income, smoking status, disease status, and laboratory test results. Body mass index (BMI) is determined by dividing an individual’s weight in kilograms by their height in meters squared. The classification of racial/ethnic groups encompasses non-Hispanic whites, non-Hispanic blacks, other Hispanic categories, Mexican Americans, and additional racial/ethnic divisions, with the income-to-poverty line ratio serving as a measure of household income. Education level is classified as below ninth grade, ninth through 11th grade, high school degree, college or associate of arts degree, college degree or above. Hypertension is characterized by a systolic blood pressure (SBP) exceeding 140 mmHg, a diastolic blood pressure (DBP) over 90 mmHg, or a self-reported diagnosis of hypertension. Clinical measures such as HbA1c, TG, HDL, and LDL were measured in the NHANES laboratory.

### Statistical analysis

For statistical analysis using Python 3.8.8 and R 4.2.1, a bilateral P < 0.05 was considered statistically significant. For the description of baseline variables, we grouped and summarized them according to the OBS and CDAI quantiles. Variables that follow a normal distribution are expressed as the mean ± standard deviation, while categorical variables are shown as counts (percentages). To evaluate differences in baseline characteristics among three score groups (quantile groups), univariate ANOVA was utilized for continuous variables, and the Pearson Chi-square test was applied to categorical variables. The analysis of the association between OBS, CDAI, and DR was conducted in three primary phases. Step 1: logistic regression analysis, model 1, unadjusted covariates; model 2, adjusted for age and gender; model 3, adding household income, race, and education level to model 2; model 4, adding all covariables to the model. Multiple imputation was performed for covariates with missing values using the KNNImputer method of the sklearn package. Step 2: the four-node restricted cubic spline (RCS) nested in logistic regression was used to fit the smooth curve to check whether there was a nonlinear relationship. Step 3: stratified analysis and interaction tests were performed.

### Ethics statement

The research design and methodologies received approval from the National Center for Health Statistics Ethics Review Board, and all participants provided written consent. Our study was deemed exempt from institutional oversight as it utilized secondary data analysis derived from the National Health and Nutrition Examination Survey.

## Results

### Baseline characteristics

In the OBS investigation, we conducted a statistical analysis of the initial traits of the participants, with findings presented in Table 1. Our analysis revealed a negative correlation between OBS levels and the incidence of DR in diabetic patients, indicating that an increase in OBS is linked to a reduced risk of developing DR. Furthermore, we noted several other factors of relevance, including the percentage of people in the highest OBS group of the highly educated subjects (with a college degree or more) is significantly higher than that in the less educated subjects (with a high school degree or less); non-Hispanic whites and blacks made up the vast majority of participants (69%), and non-Hispanic whites have a significantly higher percentage in the highest OBS group than other races; the mean age of subjects in the highest OBS group was lower (60.38 ± 14.24 y) than those in the lowest OBS group; household income is positively correlated with OBS, that is, the higher the household income, the higher the OBS.

**Table 1:** The baseline characteristics by tertiles of the OBS: National Health and Nutrition Examination Survey 2007–2008, 2011–2012.

	All	Q1 (≤ 14.00)	Q2 (14.00 to 23.00)	Q3 (> 23.00)	p value
N (%)	1287	440 (34.19)	467 (36.29)	380 (29.53)	
Age, years, mean ± SD	61.58 ± 13.54	62.81 ± 13.04	61.38 ± 13.35	60.38 ± 14.24	0.035*
Gender, n (%)					
Male	648 (50.35)	162 (36.82)	226 (48.39)	260 (68.42)	< 0.001^#^
Female	639 (49.65)	278 (63.18)	241 (51.61)	120 (31.58)	
Race, n (%)					
Mexican American	173 (13.44)	58 (13.18)	59 (12.63)	56 (14.74)	0.001^#^
Other Hispanic	132 (10.26)	51 (11.59)	52 (11.13)	29 (7.63)	
Non-Hispanic white	469 (36.44)	147 (33.41)	156 (33.41)	166 (43.68)	
Non-Hispanic black	421 (32.71)	164 (37.27)	160 (34.26)	97 (25.53)	
Other race	92 (7.15)	20 (4.54)	40 (8.56)	32 (8.42)	
Household income, mean ± SD	2.15 ± 1.43	1.81 ± 1.28	2.16 ± 1.41	2.55 ± 1.53	< 0.001^#^
Education level, n (%)					
Less than 9th grade	249 (19.35)	110 (25.00)	80 (17.13)	59 (15.53)	< 0.001^#^
9–11th grade	259 (20.12)	112 (25.45)	93 (19.91)	54 (14.21)	
High school grade	297 (23.08)	96 (21.82)	114 (24.41)	87 (22.89)	
College or AA degree	293 (22.77)	84 (19.09)	116 (24.84)	93 (24.47)	
College graduate or above	189 (14.69)	38 (8.64)	64 (13.70)	87 (22.89)	
Hypertension, n (%)					
No	396 (30.77)	115 (26.14)	142 (30.41)	139 (36.58)	0.005^#^
Yes	891 (69.23)	325 (73.86)	325 (69.59)	241 (63.42)	
Total cholesterol (TG), mg/dL, mean ± SD	165.22 ± 123.15	159.42 ± 113.05	176.29 ± 152.47	158.35 ± 88.54	0.051*
HDL-C, mg/dL, mean ± SD	47.69 ± 13.48	47.87 ± 14.49	48.02 ± 13.29	47.08 ± 12.46	0.569*
LDL-C, mg/dL, mean ± SD	100.99 ± 28.20	100.37 ± 30.98	101.25 ± 26.95	101.38 ± 26.32	0.849*
Glycohemoglobin, %, mean ± SD	7.43 ± 1.77	7.45 ± 1.80	7.41 ± 1.75	7.42 ± 1.77	0.941*
Diabetic retinopathy (DR), n (%)					
No	1021 (79.33)	327 (74.32)	380 (81.37)	314 (82.63)	0.005^#^
Yes	266 (20.67)	113 (25.68)	87 (18.63)	66 (17.37)	

^#^The p-value is derived from the Pearson Chi-square test.

*The p-value is determined by univariate ANOVA.

In the CDAI study, we also conducted descriptive statistics on the baseline characteristics of the subjects, and the results are shown in Table 2. We found that in patients with diabetes, there was no significant negative correlation between CDAI and the occurrence of DR. In terms of education level, age, and race, we found similar trends to the OBS study, including the percentage of people in the highest CDAI group of the highly educated subjects (with a college degree or more) is significantly higher than that in the less educated subjects (with a high school degree or less); non-Hispanic whites and blacks made up the vast majority of participants (69%), and non-Hispanic whites have a significantly higher percentage in the highest CDAI group than other races; the mean age of subjects in the highest CDAI group was lower (59.68 ± 14.18 y) than those in the lowest CDAI group; household income shows a positive relationship with the CDAI score, meaning as household income rises, so does the CDAI.

**Table 2:** The baseline characteristics by tertiles of the CDAI: National Health and Nutrition Examination Survey 2007–2008, 2011–2012.

	All	Q1 (≤ − 2.01)	Q2 (− 2.01 to 0.96)	Q3 (> 0.96)	p value
N (%)	1287	429 (33.33)	429 (33.33)	429 (33.33)	
Age, years, mean ± SD	61.58 ± 13.54	62.92 ± 13.13	62.13 ± 13.10	59.68 ± 14.18	0.001*
Gender, n (%)					
Male	648 (50.35)	172 (40.09)	196 (45.69)	280 (65.27)	< 0.001^#^
Female	639 (49.65)	257 (59.91)	233 (54.31)	149 (34.73)	
Race, n (%)					
Mexican American	173 (13.44)	63 (14.69)	49 (11.42)	61 (14.22)	0.019^#^
Other Hispanic	132 (10.26)	56 (13.05)	49 (11.42)	27 (6.29)	
Non-Hispanic white	469 (36.44)	138 (32.17)	158 (36.83)	173 (40.33)	
Non-Hispanic black	421 (32.71)	146 (34.03)	142 (33.10)	133 (31.00)	
Other race	92 (7.15)	26 (6.06)	31 (7.23)	35 (8.16)	
Household income, mean ± SD	2.15 ± 1.43	1.82 ± 1.26	2.15 ± 1.41	2.49 ± 1.53	< 0.001^#^
Education level, n (%)					
Less than 9th grade	249 (19.35)	114 (26.57)	69 (16.08)	66 (15.38)	< 0.001^#^
9–11th grade	259 (20.12)	98 (22.84)	92 (21.45)	69 (16.08)	
High school grad	297 (23.08)	98 (22.84)	102 (23.78)	97 (22.61)	
College or AA degree	293 (22.77)	76 (17.72)	107 (24.94)	110 (25.64)	
College graduate or above	189 (14.69)	43 (10.02)	59 (13.75)	87 (20.28)	
Hypertension, n (%)					
No	396 (30.77)	124 (28.90)	127 (29.60)	145 (33.80)	0.244^#^
Yes	891 (69.23)	305 (71.10)	302 (70.40)	284 (66.20)	
Total cholesterol (TG), mg/dL, mean ± SD	165.22 ± 123.15	164.21 ± 118.37	167.65 ± 150.00	163.82 ± 95.14	0.882*
HDL-C, mg/dL, mean ± SD	47.69 ± 13.48	48.04 ± 14.41	48.18 ± 13.74	46.86 ± 12.17	0.290*
LDL-C, mg/dL, mean ± SD	100.99 ± 28.20	100.75 ± 31.72	100.35 ± 27.00	101.87 ± 25.55	0.713*
Glycohemoglobin, %, mean ± SD	7.43 ± 1.77	7.41 ± 1.77	7.38 ± 1.79	7.49 ± 1.76	0.648*
Diabetic retinopathy (DR), n (%)					
No	1021 (79.33)	331 (77.16)	344 (80.19)	346 (80.65)	0.389^#^
Yes	266 (20.67)	98 (22.84)	85 (19.81)	83 (19.35)	

^#^The p-value is derived from the Pearson Chi-square test.

*The p-value is determined by univariate ANOVA.

### Association between the OBS/CDAI and DR

**Figure 2:**
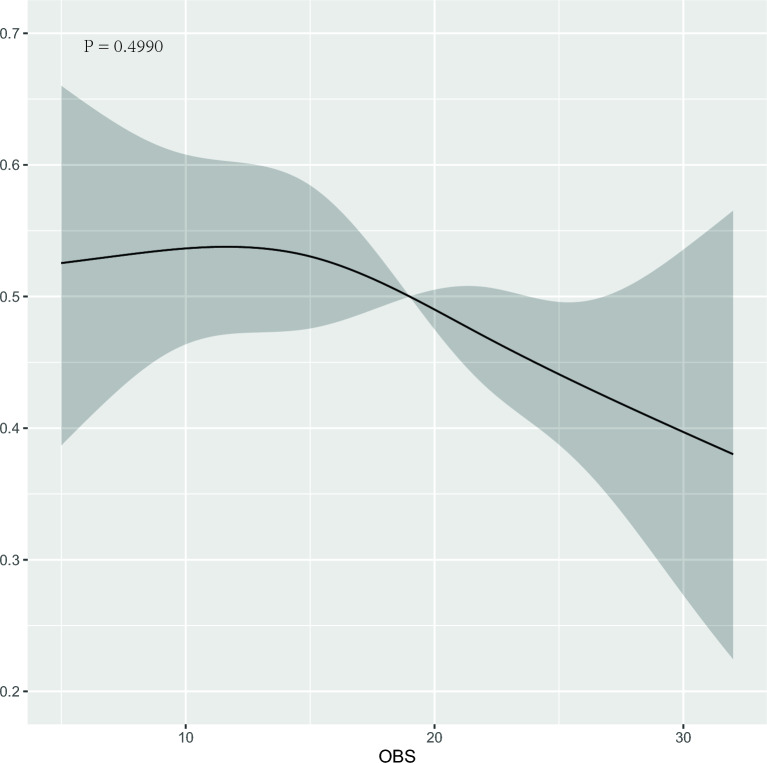
Predicted spline curves for the associations between the OBS and DR among the overall participants using restricted cubic spline regression models.

**Figure 3:**
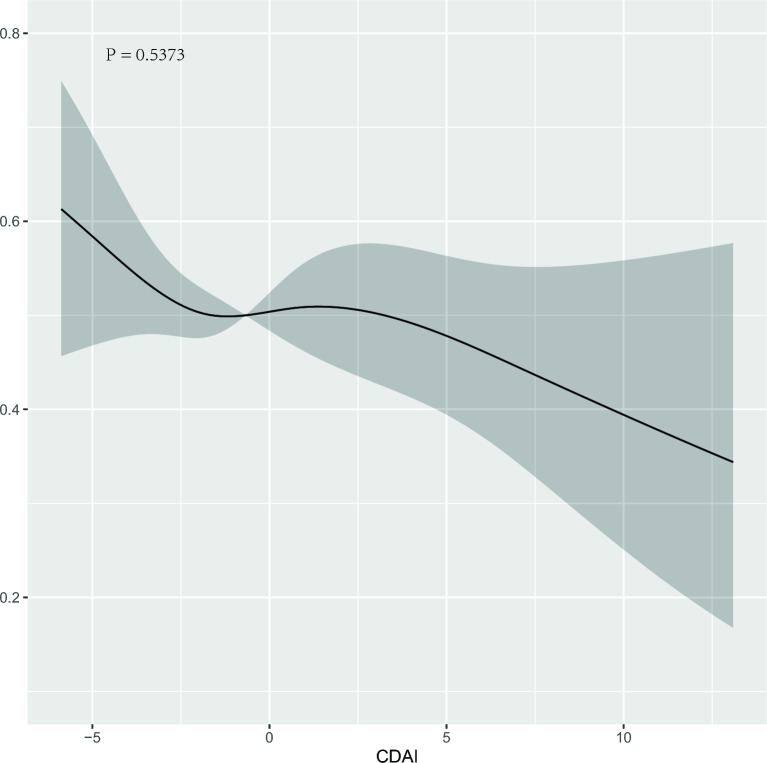
Predicted spline curves for the associations between the CDAI and DR among the overall participants using restricted cubic spline regression models.

In order to explore the relationship between OBS and CDAI and DR, we established four multiple logistic regression models respectively, and adjusted each model. Table 3 shows the correlation analysis between OBS and DR. In the model considering OBS, we controlled for confounding factors such as age, gender, race, household income, education level, hypertension, LDL, HDL, TG, and HbA1c in model 4, and found that when OBS is a continuous variable, the OR (95% CI) were [0.976 (0.956–0.996), p = 0.020], indicating that the risk of DR was reduced by 2.4% for every unit increase in OBS. In addition, we also grouped OBS by quantile (≤ 14.00, 14.00–23.00, > 23.00) and found that compared with the reference group of the lowest quantile group, the OR of the highest quantile group and its 95% confidence interval were [0.644 (0.444–0.930)]. The results showed that subjects with the highest OBS had a 35.6% lower risk of developing DR than those with the lowest OBS, and the relationship between OBS and DR showed a significant linear trend (P-trend = 0.013). Table 4 shows the correlation analysis between CDAI and DR. In the model considering CDAI, we controlled for confounding factors such as age, gender, race, household income, education level, BMI, alcohol consumption, smoking, exercise, hypertension, LDL, HDL, TG, and HbA1c in model 4, and found that when CDAI was used as a continuous variable, the OR and its 95% CI were [0.963 (0.924–1.001), p = 0.065]. The results showed that there was no statistically significant negative correlation between CDAI and the occurrence of DR. We also grouped CDAI according to quantile (≤ − 2.01, − 2.01–0.96, > 0.96), and found that compared with the reference group of the lowest quantile group, the OR of the highest quantile group and its 95% confidence interval were [0.855 (0.600–1.216)]. This result was not statistically significant, and the relationship between CDAI and DR did not show a significant linear trend (P-trend = 0.375). Finally, we also used restricted cubic spline (RCS) logistic regression to test the nonlinear relationship between OBS/CDAI and DR (Figs. 2, 3), the results of OBS are not statistically significant (p = 0.499), as the result of CDAI (p = 0.537).

**Table 3:** OR estimates for associations between dietary/lifestyle OBS and DR.

	Model I		Model II		Model III		Model IV	
	OR (95% CI)	p-value	OR (95% CI)	p-value	OR (95% CI)	p-value	OR (95% CI)	p-value
OBS (per unit increase)	0.973 (0.955–0.992)	0.005	0.970 (0.951–0.989)	0.002	0.975 (0.956–0.995)	0.015	0.976 (0.956–0.996)	0.02
OBS (tertiles)								
Q1	Ref		Ref		Ref		Ref	
Q2 (OR 95% CI)	0.663 (0.482–0.908)	0.011	0.650 (0.471–0.893)	0.008	0.665 (0.480–0.919)	0.014	0.670 (0.482–0.929)	0.017
Q3 (OR 95% CI)	0.608 (0.431–0.853)	0.004	0.571 (0.400–0.812)	0.002	0.622 (0.430–0.894)	0.011	0.644 (0.444–0.930)	0.02
P-trend		0.003		0.001		0.007		0.013

Model I: no covariates were adjusted. Model II: adjusted for age and gender. Model III: adjusted for age, gender, race, household income and education level. Model IV: adjusted for age, gender, race, household income, education level, hypertension, LDL, HDL, TG and HbA1c.

**Table 4:** OR estimates for associations between CDAI and DR.

	Model I		Model II		Model III		Model IV	
	OR (95% CI)	p-value	OR (95% CI)	p-value	OR (95% CI)	p-value	OR (95% CI)	p-value
CDAI (per unit increase)	0.961 (0.924–0.997)	0.038	0.957 (0.919–0.994)	0.028	0.964 (0.925–1.002)	0.068	0.963 (0.924–1.001)	0.065
CDAI(tertiles)								
Q1	Ref		Ref		Ref		Ref	
Q2	0.835 (0.601–1.157)	0.279	0.832 (0.598–1.155)	0.272	0.855 (0.612–1.192)	0.356	0.865 (0.616–1.212)	0.399
Q3	0.810 (0.582–1.125)	0.21	0.797 (0.567–1.117)	0.188	0.857 (0.605–1.212)	0.383	0.855 (0.600–1.216)	0.383
P-trend		0.206		0.182		0.373		0.375

Model I: no covariates were adjusted. Model II: adjusted for age and gender. Model III: adjusted for age, gender, race, household income and education level. Model IV: adjusted for age, gender, race, household income, education level, BMI, alcohol, smoke, sport, hypertension, LDL, HDL, TG and HbA1c.

### Stratified analysis

In order to test whether the relationship between CDAI and OBS and DR is heterogeneous in different populations, we conducted stratified analysis for the five components of CDAI and OBS respectively, and the results are shown in Table 5. In the stratified analysis of OBS, we found significant interactions only in the age subgroup (P-interaction = 0.025), suggesting that the relationship between OBS and DR differs in subjects of different ages. Specifically, the protective effect of increased OBS against DR was more pronounced in subjects aged ≥ 60 years (OR: 0.969; 95% CI 0.944–0.994); in the stratified analysis of CDAI, we also found significant interactions only in the age subgroup (P-interaction = 0.007), suggesting that the relationship between CDAI and DR is different in subjects of different ages. Specifically, the protective effect of increased CDAI against DR was more pronounced in subjects aged ≥ 60 years (OR: 0.943; 95% CI 0.893–0.992). While no significant interaction was detected between gender and OBS/CDAI, baseline data suggests a higher proportion of males with high OBS and CDAI, and females with low OBS and CDAI. Regression analysis conducted separately for males and females revealed a negative correlation between OBS/CDAI and the occurrence of DR exclusively in males, with no significant association found in females, indicating that the effects of OBS/CDAI may vary across genders. In addition, we found no significant interaction between CDAI and OBS and DR in education level, race, and household income subgroups.

**Table 5:** Stratified analysis of OBS/CDAI on the occurrence of DR by age, gender, education level, race and income.

Subgroup	No. of participants (percent, %)	OBS		CDAI	
		OR (95% CI)	P for interaction	OR (95% CI)	P for interaction
< Age (years)					
60	492 (38.23%)	0.987 (0.953–1.024)	0.025	0.992 (0.928–1.056)	0.007
≥ 60	795 (61.77%)	0.969 (0.944–0.994)		0.943 (0.893–0.992)	
Gender					
Male	648 (50.35%)	0.967 (0.941–0.995)	0.912	0.943 (0.893–0.992)	0.595
Female	639 (49.65%)	0.978 (0.948–1.010)		0.980 (0.916–1.045)	
Education level					
Less than 9th grade	249 (19.35%)	0.967 (0.924–1.011)	0.803	0.936 (0.847–1.019)	0.695
9–11th grade (includes 12th grade with no diploma)	259 (20.12%)	0.975 (0.931–1.020)		0.923 (0.833–1.014)	
High school grad/GED or equivalent	297 (23.08%)	0.978 (0.935–1.024)		1.026 (0.940–1.116)	
Some college or AA degree	293 (22.77%)	0.976 (0.931–1.024)		0.973 (0.888–1.060)	
College graduate or above	189 (14.69%)	0.967 (0.907–1.030)		0.924 (0.812–1.034)	
Race					
Mexican American	173 (13.44%)	0.973 (0.915–1.034)	0.840	0.992 (0.864–1.131)	0.571
Other Hispanic	132 (10.26%)	1.072 (1.001–1.151)		1.153 (0.995–1.343)	
Non-Hispanic white	469 (36.44%)	0.963 (0.929–0.997)		0.967 (0.900–1.035)	
Non-Hispanic black	421 (32.71%)	0.962 (0.927–0.997)		0.921 (0.855–0.988)	
Other race—including multi-racial	92 (7.15%)	0.972 (0.874–1.079)		0.852 (0.671–1.027)	
Household income					
< 3	968 (75.21%)	0.983 (0.960–1.006)	0.268	0.978 (0.931–1.025)	0.512
≥ 3	319 (24.79%)	0.950 (0.909–0.993)		0.927 (0.850–1.000)	

## Discussion

Using 1287 participants in the NHANES cohort, a cross-sectional study was conducted to investigate the association of OBS and CDAI as dietary and lifestyle antioxidant indicators with DR. In multivariate logistic regression model 4, we found a significant negative correlation between OBS and DR (OR: 0.976; 95% CI 0.956–0.996), suggesting that the higher the OBS, the lower the risk of DR, but there was no statistically significant negative correlation between CDAI and DR (p > 0.05).In stratified analysis, we also found that age was a significant interaction factor in the relationship between OBS and CDAI and DR (P-interaction = 0.025), that is, the protective effect of OBS and CDAI on DR was more significant in subjects aged ≥ 60 years.

The underlying biological mechanisms of OBS and CDAI and DR are unclear, but they may be related to oxidative stress. Oxidative stress is characterized by an imbalance between free radicals and antioxidants, leading to cellular and tissue damage^17^. The secondary release of oxygen-free radicals in the inflammatory state may aggravate the burden of oxidative stress^18,19^. Antioxidants can mitigate the effects of oxidative stress by donating electrons to neutralize free radicals, and there may be a synergistic effect between multiple antioxidants. Dietary antioxidants can combat oxidative stress and fulfill an antioxidant role through their bioactive molecules. Antioxidant nutrients may help reduce the risk of diabetes-related complications due to oxidative stress^20^. Oxidative stress can contribute to or worsen metabolic disorders resulting from hyperglycemia, such as heightened polyol and hexosamine pathways, activation of protein kinase C (PKC), and the formation of advanced glycation end products (AGEs). In addition, high blood sugar can also inhibit the antioxidant defense system through epigenetic modification, causing an imbalance between free radical clearance and production^21^. Excess free radicals can induce mitochondrial dysfunction, apoptosis, inflammation, lipid peroxidation, and structural and functional changes in the retina^22,23^. Diabetes can also influence the levels of copper, zinc, magnesium, and lipid peroxidation. Mineral metabolism disruptions are more pronounced in diabetic patients with complications^24^. In addition, studies have confirmed that AGEs are important indicators of metabolic disorders and oxidative stress in diabetic patients, and are strongly associated with cardiovascular disease and multiple neurodegenerative diseases^10^. Due to the increased levels of AGEs in the elderly, the therapeutic strategy of using antioxidants to clear AGEs may have better efficacy^25^. Metal elements such as Cu and Zn and vitamins A, B, C and D can reduce blood sugar by regulating related antioxidant pathways and related enzyme pathways, which also suggests why an antioxidant diet is effective in reducing the incidence of DR. Estrogen is crucial in metabolism. This is based on the synergistic effect between different forms of estrogen, which maintains glucose homeostasis while preventing oxidative damage as well as lipid-induced inflammation. This also results in high OBS and CDAI affecting women less significantly than men^26^.

Prior research has established that OBS exhibits a negative correlation with the incidence of type 2 diabetes^27^, and similar findings have been reported for CDAI with regard to diabetes prevalence^20^. Furthermore, the link between oxidative stress and DR^22^, along with the potential involvement of disrupted copper (Cu) and zinc (Zn) metabolism in the development of diabetes and its complications, has been explored^28^. The consumption of Cu, Zn, and other trace elements is believed to be inversely related to DR risk^29^. Specifically, a higher intake of zinc has been shown to mitigate the glucose-increasing effect of the rs11558471 SLC30A8 (zinc transporter) gene variant^30^. Cu-dependent lysyl oxidase is an enzyme necessary for the assembly of the extracellular matrix and it has an important catalytic role in this regard^31^. In addition, increased intake of vitamins A, B1, C, and D is thought to have antioxidant effects and reduce the risk of DR. Implementing strategies centered on neuroprotection is considered key to treating the early stages of diabetes^32^. In addition, previous studies have shown a bidirectional relationship between major metabolic abnormalities induced by diabetes and oxidative stress^33^. All these studies suggest that antioxidant and pro-oxidant intake may influence the development of diabetes and its associated complications, which provides theoretical support for the present study.

The present study pioneered the use of both OBS and CDAI to assess the association between dietary and lifestyle antioxidant capacity and diabetic retinopathy (DR). The combination of OBS and CDAI helps to overcome the inherent shortcomings of using a single antioxidant or total antioxidant capacity (TAC) metric, and to provide a more comprehensive reflection of recent antioxidant intake from a variety of dietary sources. This approach reduces the inaccuracies and randomization associated with reliance on a single indicator. However, the cross-sectional nature of this study also presents limitations, particularly the inability to determine causal relationships between OBS, CDAI, and DR, or to completely rule out the impact of potential confounding variables. Despite adjustments for numerous covariates and performing stratified analyses to account for confounders, the possibility of unmeasured or overlooked confounding factors remains. Furthermore, the data, sourced from NHANES, depicts the U.S. population, limiting its applicability to more racially diverse populations. While the study excludes minors and acknowledges that type 2 diabetes significantly predominates among adult diabetes cases, the absence of data on insulin-related antibodies in NHANES constrains our ability to definitively distinguish between type 1 and type 2 diabetes.

## Conclusion

Using 1287 participants from the NHANES cohort, we investigated the association of OBS and CDAI as dietary and lifestyle antioxidant markers with DR. Multivariate logistic regression analysis showed that OBS was significantly negatively correlated with DR, but there is no statistically significant negative correlation between CDAI and DR, indicating that the higher the antioxidant capacity of diet and lifestyle, the lower the risk of DR. Stratified analysis also found that age was a significant interaction factor in the relationship between OBS and CDAI and DR, that is, the protective effect of OBS and CDAI on DR was more significant in subjects aged 60 years or older. The findings of this study suggest that adopting an antioxidant-rich diet and lifestyle could be a significant contributor to the prevention of DR.

## Data Availability

National Health and Nutrition Examination Survey (NHANES) (https://www.cdc.gov/nchs/nhanes/index.htm) provides data for the survey.

## Abbreviations

DR: Diabetic retinopathy
OBS: Oxidation balance score
CDAI: Combined dietary antioxidant index
NHANES: National health and nutrition examination survey
RCS: Restricted cubic splines
BMI: Body mass index
MET: Metabolic equivalent
AP: Activity intensity
PKC: Protein kinase C
AGEs: Advanced glycation end products
LDL: Low-density lipoprotein
HDL: High-density lipoprotein
OR: Odds ratio
CI: Confidence interval
TG: Triglyceride
SBP: Systolic blood pressure
DBP: Diastolic blood pressure
TAC: Total antioxidant capacity
HbA1c: Hemoglobin A1C
